# ON‐rep‐seq as a rapid and cost‐effective alternative to whole‐genome sequencing for species‐level identification and strain‐level discrimination of *Listeria monocytogenes* contamination in a salmon processing plant

**DOI:** 10.1002/mbo3.1246

**Published:** 2021-11-15

**Authors:** Gunn Merethe Bjørge Thomassen, Lukasz Krych, Susanne Knøchel, Lisbeth Mehli

**Affiliations:** ^1^ Department of Biotechnology and Food Science Norwegian University of Science and Technology (NTNU) Trondheim Norway; ^2^ Department of Food Science University of Copenhagen Frederiksberg Denmark

**Keywords:** foodborne pathogen, *Listeria monocytogenes*, ON‐rep‐seq, Oxford Nanopore technology, whole‐genome sequencing

## Abstract

Identification, source tracking, and surveillance of food pathogens are crucial factors for the food‐producing industry. Over the last decade, the techniques used for this have moved from conventional enrichment methods, through species‐specific detection by PCR to sequencing‐based methods, whole‐genome sequencing (WGS) being the ultimate method. However, using WGS requires the right infrastructure, high computational power, and bioinformatics expertise. Therefore, there is a need for faster, more cost‐effective, and more user‐friendly methods. A newly developed method, ON‐rep‐seq, combines the classical rep‐PCR method with nanopore sequencing, resulting in a highly discriminating set of sequences that can be used for species identification and also strain discrimination. This study is essentially a real industry case from a salmon processing plant. Twenty *Listeria monocytogenes* isolates were analyzed both by ON‐rep‐seq and WGS to identify and differentiate putative *L*. *monocytogenes* from a routine sampling of processing equipment and products, and finally, compare the strain‐level discriminatory power of ON‐rep‐seq to different analyzing levels delivered from the WGS data. The analyses revealed that among the isolates tested there were three different strains. The isolates of the most frequently detected strain (*n* = 15) were all detected in the problematic area in the processing plant. The strain level discrimination done by ON‐rep‐seq was in full accordance with the interpretation of WGS data. Our findings also demonstrate that ON‐rep‐seq may serve as a primary screening method alternative to WGS for identification and strain‐level differentiation for surveillance of potential pathogens in a food‐producing environment.

## Graphical abstract

Strain discrimination of *Listeria monocytogenes* detected in a food‐producing environment is important as it can reveal if the positive *L. monocytogenes* sample is due to a persistent strain or a transient strain. Here we demonstrate how Oxford Nanopore Technology‐based rep‐PCR amplicon sequencing (ON‐rep‐seq) can serve as an accurate and efficient method for strain discrimination of 20 *L. monocytogenes* detected in a salmon processing plant. The clustering pattern demonstrated by ON‐rep‐seq was in full accordance with that achieved by whole‐genome sequencing.

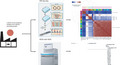

## 1 INTRODUCTION

Intra‐species variability exists in the bacterial genome (Abee et al., 2016; Sela et al., 2018) and therefore strain‐level discrimination of pathogens is a key factor for the identification and subsequent elimination of a contamination source at a food processing plant. The significance of *Listeria monocytogenes* as a foodborne pathogen is well documented (Buchanan et al., 2017; Farber & Peterkin, 1991; Gandhi & Chikindas, 2007), and through the years different microbial typing methods, more or less labor‐intensive, have been used to identify and differentiate this pathogen at the strain level (Jadhav et al., 2012; Wiedmann, 2002). During the last decades, development in sequencing technologies and whole‐genome sequencing (WGS) has rapidly been changing bacterial strain identification analysis in the food industry. WGS is now becoming a more available and affordable molecular tool and is proposed to be the new primary typing tool for strain identification of *L*. *monocytogenes* (Moura et al., 2017). It has already been successfully used to investigate and characterize outbreaks of listeriosis (Jackson et al., 2016; Kvistholm Jensen et al., 2016; Schjørring et al., 2017). *L*. *monocytogenes* is a highly heterogeneous, omnipresent, psychrotolerant pathogen (Moura et al., 2016), able to survive and persist in food processing plants for years (Fagerlund et al., 2016). The possibility of *L*. *monocytogenes* contamination in food products from residual cells in the equipment represents a serious concern, especially in the ready‐to‐eat (RTE) food industry (EFSA, 2018; Fonnesbech Vogel et al., 2001). Many food processing plants have therefore implemented a comprehensive testing regime to detect this pathogen in raw materials, processing environment, equipment, and food products (Carpentier & Léna, 2012; EuropeanCommision, 2005). Whenever a food processing plant experience frequent detection of *L*. *monocytogenes* it raises the question of whether the contamination is due to a persistent strain or transient strains. Identification at the strain level and source tracking are therefore crucial to recognize possible “hot spots” for accommodating the pathogen.

Sequence‐based typing, and in particular whole genome sequencing (WGS), are proposed to replace pulse‐field gel electrophoresis (PFGE) as the primary typing method for *L*. *monocytogenes* (Moura et al., 2017) as well as for other foodborne pathogens (Oakeson et al., 2017). Nevertheless, PFGE, MLST (multilocus sequence type), and other typing methods will remain relevant techniques for smaller laboratories also in years to come (Neoh et al., 2019) because of the significant investments necessary to implement WGS in strain typing (Nouws et al., 2020).

In theory, WGS can differentiate strains on a single nucleotide level and it has a resolution superior to PFGE and MLST (Salipante et al., 2015; Stasiewicz et al., 2015), and is gaining support in both outbreak investigation, surveillance, and source tracking of pathogenic bacteria (Nadon et al., 2017; Van Walle et al., 2018; Zhang et al., 2020). So, WGS analysis generated with short‐read technology offered by Illumina sequencing platforms is cost‐effective, accurate, and offers a low sequencing cost per base however with the limitations of short reads and challenging genome assembly (Kwong et al., 2015; Xu et al., 2020). Additional important drawbacks of the WGS as a molecular tool for institutions lacking bioinformatics infrastructure and expertise is the comprehensive data analysis and data interpretation (Oakeson et al., 2017). There is, however, a variety of WGS data analysis pipelines available (Jagadeesan, Baert, et al., 2019; Quainoo et al., 2017), ranging from methods that require extensive bioinformatics expertise to commercial software packages which can be challenging to use (Amézquita et al., 2020; Jagadeesan, Gerner‐Smidt, et al., 2019). Nevertheless, studies have shown that source tracking with WGS data from *L*. *monocytogenes* was possible from these platforms with default settings (Jagadeesan, Gerner‐Smidt, et al., 2019; Oakeson et al., 2017).

The third‐generation sequencing technologies allow for long sequencing reads of single molecules which simplifies the reconstruction of the molecules and de novo assembly of genomes. One of the cheapest (~$1000) and most commonly used is a MinION sequencer commercialized in 2014 by Oxford Nanopore Technologies (ONT) (Jain et al., 2015; Loman & Watson, 2015). In its early days, this technology had limitations due to the high error rate and relatively low throughput (Kilianski et al., 2015; van Dijk et al., 2018). Since then the technology has matured significantly with a reduced error rate and higher throughput (Karst et al., 2021). Considering ONT's latest release, Flongle, which is a $90 adapter for the MinION transportable sequencing platform, the sequencing cost is now considerably decreased.

The classical fingerprinting method, repetitive sequence‐based PCR (rep‐PCR) was introduced in 1991 by Versalovic et al. (1991) and has been shown to have equal discriminatory power as PFGE for subtyping *Listeria monocytogenes* (Chou & Wang, 2006). By combining rep‐PCR with the sequencing of the amplicons with the ONT sequencing platform Krych et al. (2019) presented a new method called ON‐rep‐seq. This method combines the discriminative power of rep‐PCR fingerprinting with access to the sequence information for each DNA fragment which we earlier only knew as bands on a gel. This gives a set of highly discriminating sequences which allows for accurate taxonomic identification and in many cases strain‐level differentiation (Krych et al., 2019).

This study aimed to explore the use of ON‐rep‐seq as (1) a screening method in a real industry case for identification and differentiation of putative *L*. *monocytogenes* isolated during routine sampling of processing equipment and products and (2) to evaluate the strain level discrimination results with WGS.

## 2 MATERIALS AND METHODS

### 2.1 Sampling in processing plant and preparation of isolates

Routine sampling in the salmon processing plant was performed according to the company's guidelines. Environmental testing was performed both at fixed and rotational sampling points every day, before, during, and after the processing of the salmon. Analysis of the samples was performed at the in‐house laboratory of the processing plant following the iQ‐Check *Listeria* spp. kit (Bio‐Rad) procedure. All PCR‐positive samples were plated on Rapid’L.mono agar plates (Bio‐Rad). From all plates that contained colonies with typical characteristics of *L*. *monocytogenes* colony, the material was frozen at −20℃ and stored in the Microbank^TM^ system (Pro‐Lab Diagnostics) before being transported to NTNU and further stored at −80℃. Two gutting machine lines repeatedly tested positive for *L*. *monocytogenes* and therefore, 20 isolates deriving from different time points and places on these lines were selected for further investigations (Table 1).

**TABLE 1:** Sampling points and sampling dates of the 20 *Listeria* isolates from the two gutting machines with frequently positive *L*. *monocytogenes* samples and downstream in the processing lines. The presumptive identifications from the processing plant in‐house laboratory are listed

Isolate ID	Sampling point	Sampling date	ID Rapid’L.mono
SL3.179	Gutting machine 3	28.06.2019	*L. monocytogenes*
SL3.189	Gutting machine 3	08.07.2019	*L. monocytogenes*
SL3.212	Gutting machine 3	31.07.2019	*L. monocytogenes*
SL3.296	Gutting machine 3	23.10.2019	*L. monocytogenes*
SL6.141	Gutting machine 6	21.05.2019	*L. monocytogenes*
SL6.206	Gutting machine 6	25.07.2019	*L. monocytogenes*
SL6.212	Gutting machine 6	31.07.2019	*L. monocytogenes*
SL6.218	Gutting machine 6	06.08.2019	*L. monocytogenes*
HK1.329h	Head and tail cutter 1	25.11.2019	*L. monocytogenes*
HK1.329v	Head and tail cutter 1	25.11.2019	*L. monocytogenes*
HK1.337	Head and tail cutter 1	03.12.2019	*L. monocytogenes*
HK3.297	Head and tail cutter 3	24.10.2019	*L. monocytogenes*
HK3.331	Head and tail cutter 3	27.11.2019	*L. monocytogenes*
HK3.357	Head and tail cutter 3	23.12.2019	*L. monocytogenes*
PK.141	Packaging department	21.05.2019	*L. monocytogenes*
F1K1.353	Filleting machine 1 quality scanner1	19.12.2019	*L. monocytogenes* ^a^
F1K2.353	Filleting machine 1 quality scanner 2	19.12.2019	*L. monocytogenes*
FS.171	Fillet salmon	20.06.2019	*L. monocytogenes*
SwF1.296	Swab fillet	23.10.2019	*L. monocytogenes* ^a^
SwF1.357	Swab fillet	23.12.2019	*L. monocytogenes* ^a^

^a^ Inconclusive, suspected to be *L*. *monocytogenes*.

Upon analysis, the isolates were propagated on Brain Heart Infusion agar (BHIA; CM1136) and repropagated at a minimum twice. Their growth and appearance on Brilliance Listeria Differential agar (BLA; CM1080) was investigated after incubation at 37^o^C for 24 ± 2 h.

Note, DNA extraction was performed by using the Genomic Micro AX Bacteria+ Gravity‐kit (102–100 M, A&A BIOTECHNOLOGY) according to the manufacturer's procedure. The RNAse treatment was included in the procedure. The DNA was eluted in the neutralized elution buffer. Also, DNA quality was checked on agarose gel and DNA concentrations were estimated by spectrophotometric measurement using BioTek PowerWave XS, Take3 plate, and Gen5 2.0 software. DNA (30 µl, ~40 ng/µl) was sent on ice with overnight shipment to Novogene UK Sequencing laboratory and another 30 µl (~40 ng/µl) of DNA was subjected to ON‐rep‐seq sequencing at the University of Copenhagen, Denmark.

### 2.2 Whole‐genome sequencing

#### 2.2.1 Library construction and sequencing details

At the sequencing laboratory, DNA purity and integrity were again controlled and accurate DNA concentration was measured by Qubit^®^ 3.0 fluorometer quantification. The genomic DNA was randomly sheared into fragments of about 350 bp and library construction was done by using the NEBNext^®^ DNA Library Prep Kit. End repairing, dA‐tailing, and ligation of NEBNext^®^ adapter were done before the fragments were PCR enriched by P5 and indexed P7 oligos. Purification and quality check of the products was performed before sequencing. The sequencing strategy was paired‐end sequencing with a read length of 150 bp at each end, performed on an Illumina^®^ NovaSeq^TM^ 6000 sequencing platform.

Base‐calling was done with CASAVA v1.8 software and the raw read dataset was subject to quality filtering. Paired reads containing either adapter contamination, more than 10% uncertain nucleotides or reads with low‐quality nucleotides (base quality *Q* ≤ 5) constituting more than 50% of either read, was removed to obtain high‐quality reads.

#### 2.2.2 Genomic characterization based on WGS data

The whole‐genome sequences were analyzed by using the online web‐based tools developed by the Center for Genomic Epidemiology (CGE, 2020). The high‐quality reads from Illumina PE150 sequencing were used as templates and uploaded to the CGE server. The typing tool KmerFinder (Clausen et al., 2018; Hasman et al., 2014; Larsen et al., 2014) was used to identify the species based on Kmers (length = 16 bases), while MLST 2.0 (Larsen et al., 2012), was used to determine the sequence type based on the seven conventional MLST loci. For the 17 isolates identified as *L*. *monocytogenes* the MLST configuration *Listeria monocytogenes* was chosen, and for the three isolates identified as *L*. *innocua*, the MLST configuration, *Listera* was chosen.

Average Nucleotide Identity (ANI) is a measure used to compare the genome sequences of two prokaryotic organisms and calculate the ANI value. Here, the online ANI Calculator from ChunLab (Yoon et al., 2017), based on the OrthoANI algorithm, was used to do pairwise comparisons of all the isolates in the dataset.

To show the relationship between the *L*. *monocytogenes* isolates a phylogenetic tree based on SNPs was constructed using the CGE webtool CSI Phylogeny 1.4 (Kaas et al., 2014). Three reference genomes were included in the tree (Table 2). To give a better visualization the result file in Newick format was uploaded to another web tool, iTol (Letunic & Bork, 2019). The phylogenetic tree was rooted at the reference strain *L*. *monocytogenes* EGD‐e.

**TABLE 2:** Overview of the seven reference genomes that are used in the different analyses in this study

Reference strain	GenBank accession number	Which analyses included in			
		ON‐rep‐seq	WGS	SNP phylogeny	Ortho ANI
*Listeria monocytogenes* EGD‐e	GCA_000196035.1_ASM19603v1	X	X	X	X
*Listeria innocua* Clip11262	GCF_000195795.1_ASM19579v1	X	X		X
*Listeria monocytogenes* LO28	GCA_000168675.1_ASM16867v1	X			
*Listeria monocytogenes* N53‐1	GCA_000382945.1_ASM38294v1	X			
*Listeria monocytogenes* 12067	NA	X			
*Listeria monocytogenes R479a*	GCF_000613085.1			X	X
*Listeria monocytogenes T1‐037*	GCF_003002675.1			X	X

Further on, genotypic characterization and phenotypic predictions were made on acquired antimicrobial resistance genes using ResFinder 3.2 (Zankari et al., 2012), virulence‐associated genes using VirulenceFinder 2.0 (Joensen et al., 2014) with default settings (the threshold for ID = 90%, minimum length = 60%) and pathogenic genes using PathogeneFinder 1.1 (Cosentino et al., 2013) for bacteria in the phylum Firmicutes. Detection of plasmids was performed using the online web tool PlasmidFinder 1.2 (Carattoli et al., 2014) for Gram‐positive bacteria with the following settings: threshold for minimum identity = 80% and minimum coverage = 60%. To investigate if any of the isolates carried a truncated *inlA* gene, the sequences of each isolate's *inlA* gene were submitted to the NCBI webtool ORFfinder and analyzed for premature stop codons (PMSC).

#### 2.2.3 Comparison to other published isolates by NCBI Pathogen Detection

The WGS data from each isolate was submitted to NCBI SRA. Sequence data for pathogens submitted to SRA are regularly picked up by the NCBI Pathogen Detection project, assembled, and compared to all other assemblies in the same taxonomic group (NCBI, 2016). Isolates in the same SNP cluster differ with <50 SNPs and within each cluster, a phylogenetic tree is constructed based on a maximum compatibility algorithm (Cherry, 2017). The “Search and Highlight” function was used to find other isolates associated with salmon, fish, seafood, and food processing environment.

### 2.3 Oxford Nanopore Technology based rep‐PCR amplicon sequencing

#### 2.3.1 ON‐rep‐seq library preparation

The Rep‐PCR reaction mix contained 5 μl PCRBIO HiFi buffer (5×), 0.25 μl of PCRBIO HiFi Polymerase (PCR Biosystems Ltd), 4 μl of (GTG)5 primers (5 μM), 1 μl of DNA (~20 ng/μl) and nuclease‐free water to a total volume of 25 μl. The Rep‐PCR thermal conditions were as follows: Denaturation at 95°C for 5 min; 30 cycles of 95°C for 30 s, 45°C for 1 min and 62°C for 4 min; followed by final elongation at 72°C for 5 min using SureCycler 8800 (Agilent).

The barcoding Rep‐PCR reaction mix contained 12 μl of PCRBIO UltraMix (PCR Biosystems Ltd, London, United Kingdom) 2 μl of corresponding repBC primer (10 μM), 1 μl of PCR product from Rep‐PCR‐1 and nuclease‐free water to a total volume of 25 μl. Incorporation of ONT compatible adapters was performed using dual‐stage PCR where first 3 cycles provide optimal annealing of (GTG)5 regions, following 10 cycles of denaturation 5 min; 3 cycles of 95°C for 30 s, 45°C for 1 min and 62°C for 4 min; followed by 10 cycles of 95°C for 30 s, 65°C for 1 min and 72°C for 4 min and final elongation at 72°C for 5 min. After Rep‐PCR‐2 samples were pooled using 10 μl of each sample. The pooled library was cleaned with AMPure XP beads (Beckman Coulter Genomics) in volumes 100:50 μl respectively. The bead pellet was washed with 80% ethanol and re‐suspended in 100 μl of nuclease‐free water.

The pooled and bead‐purified library was measured with Qubit^®^ dsDNA HS Assay Kit (Life Technologies) and 66 ng of the library was used as an input to the End‐prep step in 1D amplicon by ligation protocol (ADE_9003_v108_ revT_18Oct2016) with one adjustment: 80% ethanol instead of 70% was used for all washing steps.

The sequencing was performed using the R9.4.1 flow cell.

#### 2.3.2 Data collection, base calling, demultiplexing, and trimming

Data were collected using Oxford Nanopore software: GridION 19.12.2 (https://nanoporetech.com). Guppy 4.4.0 toolkit was used to base call raw fast5 to fastq (https://nanoporetech.com) and demultiplex based on custom adapters.

#### 2.3.3 Correction and base location of peaks

Peaks are identified in LCp expressed as sequencing length (*x*‐axis) by the number of reads (*y*‐axis) by fitting local third order polynomials in a sliding window of size 1/50 of the *x*‐span across the *x*‐axis, followed by calculation of the first‐ and second‐order derivatives. Only peaks with intensity higher than baseline, defined as a moving boxcar (zero‐order polynomial) in a broad window (4 times the size of the window used for calculation of the derivative) are used for further analysis. The identified peaks are ordered based on the height, and a representative fragment is used for database matching.

Sequences containing quality scores (fastq files) resolved within each peak were retrieved using Cutadapt v1.15 (Martin, 2011) and corrected with Canu v1.6 (Koren et al., 2017) using the following parameters: genomeSize = 5k, minimumReadLength = 200, correctedErrorRate = 0.05, corOutCoverage = 5000, corMinCoverage = 2 and minOverlapLength = 50. The corrected reads were sorted by length and clustered by cluster_fast from VSEARCH (Rognes et al., 2016), using the following options: ‐id of 0.9, ‐minsl of 0.8, ‐sizeout, and min_cons_pct of 20. The purpose of this step is to detect structural sequence variants of similar length. Subsequently, consensus sequences were sorted by size (coverage), and those with a minimum coverage size of 50× were kept for downstream analyses. A detailed description of the LCp comparison algorithm is given in the original work (Krych et al., 2019). Kraken2 (Wood & Salzberg, 2014) metagenomic classifier was used for the classification of corrected reads.

## 3 RESULTS

### 3.1 Whole‐genome sequencing of the 20 *Listeria* isolates

The total amount of raw data generated by WGS was 23.0 GB, with the amount of raw data for each isolate varying between 1.1 GB to 1.6 GB. After filtering out low quality and adapter contaminations the amount of clean data for each isolate were between 99.91 to 99.98% of the raw data. Detailed quality metrics for each isolate are shown in Table 3. The SRA BioSample accession numbers for each isolate are also listed in the table.

**TABLE 3:** Each isolates accession BioSample number, of where both WGS and ON‐rep‐seq data are available, are listed together with sequencing quality data from WGS and ON‐rep‐seq respectively

Isolate ID	Accession number/BioSample	WGS			
		No. raw reads pairs	Effective reads (%)	Average depth (X)^a^	Coverage at least 4X (%)^b^
SL3.179	SAMN21435073	4,968,125	99.95	400.18	95.57
SL3.189	SAMN21435074	4,932,455	99.97	380.95	95.57
SL3.212	SAMN21435075	4,676,698	99.96	379.05	95.57
SL3.296	SAMN21435076	5,085,190	99.94	404.65	95.56
SL6.141	SAMN21435077	4,545,360	99.95	366.83	95.57
SL6.206	SAMN21435078	4,587,296	99.97	373.45	95.57
SL6.212	SAMN21435079	4,146,831	99.95	335.28	95.57
SL6.218	SAMN21435080	5,273,298	99.96	416.48	95.57
HK1.329h	SAMN21435081	3,975,803	99.95	327.09	95.55
HK1.329v	SAMN21435082	4,254,40	99.94	343.39	95.55
HK1.337	SAMN21435083	5,292,498	99.95	421.29	95.56
HK3.297	SAMN21435084	4,247,379	99.95	344.86	95.56
HK3.331	SAMN21435085	4,101,054	99.94	334.49	95.56
HK3.357	SAMN21435086	4,085,114	99.91	322.14	95.56
PK.141	SAMN21435087	3,656,738	99.95	294.98	95.56
F1K1.353	SAMN21435090	3,450,091	99.93	284.19	92.98
F1K2.353	SAMN21435089	4,400,199	99.98	334.92	95.13
FS.171	SAMN21435088	4,495,916	99.94	348.41	95.13
SwF1.296	SAMN21435091	3,866,277	99.92	315.47	92.98
SwF1.357	SAMN21435092	4,628,147	99.89	368.21	92.98

^a^ Average depth of mapped (against reference strain) reads at each site, calculated by the number of bases in the mapped reads dividing by size of the assembled genome.

^b^ The percentage of the assembled genome with ≥4X coverage at each site.

#### 3.1.1 Taxonomic identification reveals two different *Listeria* species

The online classifier KmerFinder predicted 17 of the isolates to be *L*. *monocytogenes*, while the prediction of the three remaining isolates was *L*. *innocua* (Table 4).

**TABLE 4:** Overview over species identification and strain discrimination obtained from ON‐rep‐seq data and identification, strain discrimination, and genotypic traits identified from WGS data. For comparison, the corresponding information for reference genomes *L*. *monocytogenes* EGD‐e and *L*. *innocua* Clip11262 are also included

Isolate ID	ON‐rep‐seq	WGS				
	Taxonomy assignment	ID KmerFinder	MLST	No. virulence genes (ID >98%)	Predicted human pathogen (prob.)	Plasmids
Reference		*L*. *monocytogenes* EGD‐e	35	87	Yes (0.812)	–
Reference		*L*. *innocua* Clip11262	–	0	Yes (0.812)	pLI100 (rep26) pLI100 (repUS65) pLI100 (rep32)
F1K1.353	*L. innocua*	*L. innocua*	–	2	Yes (0.818)	pLM33 (rep25)
F1K2.353	*L. monocytogenes*	*L. monocytogenes*	8	83	Yes (0.808)	–
FS.171	*L. monocytogenes*	*L. monocytogenes*	8	83	Yes (0.808)	–
HK1.329h	*L. monocytogenes*	*L. monocytogenes*	37	81	Yes (0.812)	–
HK1.329v	*L. monocytogenes*	*L. monocytogenes*	37	81	Yes (0.812)	–
HK1.337	*L. monocytogenes*	*L. monocytogenes*	37	81	Yes (0.812)	–
HK3.297	*L. monocytogenes*	*L. monocytogenes*	37	81	Yes (0.812)	–
HK3.331	*L. monocytogenes*	*L. monocytogenes*	37	81	Yes (0.812)	–
HK3.357	*L. monocytogenes*	*L. monocytogenes*	37	81	Yes (0.812)	–
PK.141	*L. monocytogenes*	*L. monocytogenes*	37	81	Yes (0.812)	–
SL3.179	*L. monocytogenes*	*L. monocytogenes*	37	81	Yes (0.812)	–
SL3.189	*L. monocytogenes*	*L. monocytogenes*	37	81	Yes (0.812)	–
SL3.212	*L. monocytogenes*	*L. monocytogenes*	37	81	Yes (0.812)	–
SL3.296	*L. monocytogenes*	*L. monocytogenes*	37	81	Yes (0.812)	–
SL6.141	*L. monocytogenes*	*L. monocytogenes*	37	81	Yes (0.812)	–
SL6.206	*L. monocytogenes*	*L. monocytogenes*	37	81	Yes (0.812)	–
SL6.212	*L. monocytogenes*	*L. monocytogenes*	37	81	Yes (0.812)	–
SL6.218	*L. monocytogenes*	*L. monocytogenes*	37	81	Yes (0.812)	–
SwF1.296	*L. innocua*	*L. innocua*	–	2	Yes (0.818)	pLM33 (rep25)
SwF1.357	*L. innocua*	*L. innocua*	–	2	Yes (0.818)	pLM33 (rep25)

#### 3.1.2 MLST profiling indicates two strains within 17 isolates of *L. monocytogenes*

Further differentiation of the isolates with the online typing tool MLST revealed 15 of the 17 isolates to be of sequence type (ST) 37 while the last two were of ST8 (Table 4).

**FIGURE 1:**
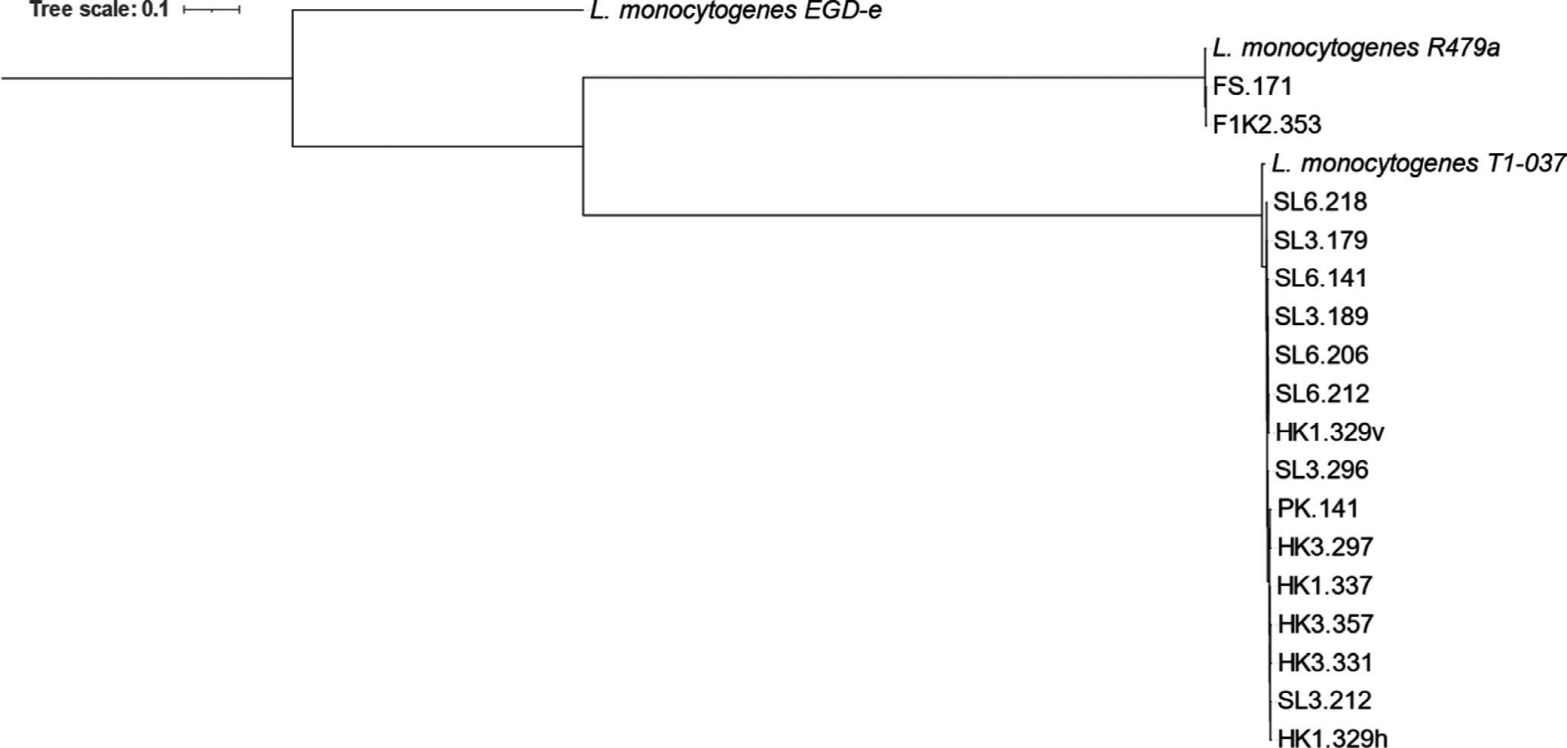
A phylogenetic tree of the 17 *L*. *monocytogenes* isolates based on SNPs. The *L*. *monocytogenes* clusters in two groups corresponding to their MLST sequence type. The two isolates F1K2.353 and FS.171 belong to ST8 and cluster together with the ST8 reference strain *L*. *monocytogenes* R479a while the other 15 isolates belong to ST37 and cluster together with the ST37 reference strain *L*. *monocytogenes* T1‐037. The tree was rooted at the reference strain *L*. *monocytogenes* EGD‐e

The phylogenetic tree based on SNPs supported the similarity of the *L*. *monocytogenes* isolates clustering in two different groups in perfect correlation with the MLST sequence type (Figure 1). The two isolates F1K2.353 and FS.171 belong to the ST8 cluster together with ST8 reference strain *L*. *monocytogenes* R479a, while the other 15 isolates belonging to ST37 cluster together with ST37 reference strain *L*. *monocytogenes* T1‐037. Both groups differentiated from *L*. *monocytogenes* EGD‐e reference strain.

#### 3.1.3 ANI analysis did not identify any strain‐level differences

**FIGURE 2:**
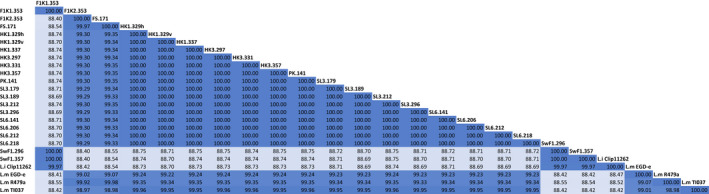
OrthoANI matrix showing the average nucleotide identity between the isolates. The ANI values between the isolates and some closely related strains are also included. Based on a cut‐off for ANI value of <99% to separate strains, this means that by the OrthoANI method the 17 *L*. *monocytogenes* isolates in this study are indistinguishable from each other. The cut‐off for species‐level discrimination is <95%. ANI values between *L*. *innocua* and *L*. *monocytogenes* strains in this study are 88–89% and give clear interspecies discrimination

Pairwise Average Nucleotide Identity (ANI) between the isolates and four reference genomes, revealed a 100.00% similarity between the 15 *L*. *monocytogenes* of ST37 (Figure 2). This indicated that they are all the same strain. Compared to the *L*. *monocytogenes* T1‐037 reference genome, also ST37, these isolates all had ANI values of 99.95%–99.96%. The ST8 isolates, F1K2.353 and FS.171 had an ANI value of 99.97% to each other and 99.92% and 99.98% respectively to the *L*. *monocytogenes* R479a (ST8) reference genome. ANI values between ST8 and ST37 isolates varied between 99.29%–99.35%, but between ST8 isolates and the reference strain *L*. *monocytogenes* T1‐037 it was down to 98.97%–98.98%. The three *L*. *innocua* isolates had ANI values of 100.00% to each other and 99.97% to *L*. *innocua* Clip11262 reference genome (Figure 2) and they cannot be differentiated from each other or *L*. *innocua* Clip11262 by this method.

#### 3.1.4 Antibiotic resistance genes, virulence genes, and pathogen genes showed no additional strain‐level differences

All the *L*. *monocytogenes* isolates in this selection carried the *fosX* gene coding for fosfomycin resistance with a sequence identity of 98.76% for ST8 isolates and 99.25% for ST37 isolates. No resistance genes included in the ResFinder 3.2 database were detected in the *L*. *innocua* isolates.

The *L*. *monocytogenes* isolates carried a large number of virulence genes. In the ST8 isolates, 21 known virulence genes with 100% ID to sequence in the database could be identified, and additionally 62 virulence genes with 98.0%–99.9% ID (Table 3). The ST37 isolates carried 24 known virulence genes with 100% ID to sequence in the database and 57 virulence genes with 98.0%–99.9% ID (Table 3).

When analyzing the *L*. *monocytogenes* isolates for a possible truncated *inlA* gene, it was confirmed that all isolates constituted a full length (2403 bp) *inlA* gene with a 98.54% and 98.21% identity for ST8 and ST37 isolates, respectively. The NCBI webtool ORFfinder reported no in‐frame premature stop codons for any of the isolates.

The selected isolates in this study, including the *L*. *innocua* isolates, were predicted to be human pathogens by the web tool PathogenFinder 1.1 with probability 0.812 for the ST37 isolates, 0.808 for ST8 isolates, and 0.818 for *L*. *innocua* isolates. However, the *prfA* gene coding for positive regulatory factor A (PrfA) in *L*. *monocytogenes*, was absent from the *L*. *innocua* isolates.

#### 3.1.5 No strain‐specific plasmids were found

When applying the default settings (95% identity, 60% coverage) in the webtool PlasmidFinder 1.2 no plasmids could be detected in the 17 *L*. *monocytogenes* isolates (Table 4). Lowering the identity cutoff to 80% enabled the detection of the rep26 sequence of pLM5578 (84% ID) (Gilmour et al., 2010) and the rep26 sequence of PLGUG1 originally isolated from *L*. *grayi* (Kuenne et al., 2010) in the *L*. *monocytogenes* ST8 isolate, F1K2.353. Interestingly the three *L*. *innocua* isolates were found to carry a plasmid with 100% similarity to plasmid pLM33 which is commonly found in food‐related lineage II *L*. *monocytogenes* strains (Canchaya et al., 2010).

#### 3.1.6 NCBI pathogen detection pipeline assigned the *L. monocytogenes* isolates in two different SNP clusters

**FIGURE 3:**
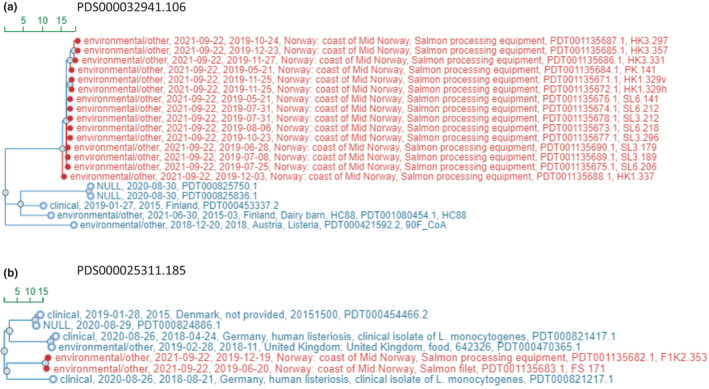
Maximum compatibility phylogenetic tree of *L*. *monocytogenes* isolates generated by NCBI Pathogen Detection pipeline. (A) shows a subtree of SNP cluster PDS000032941.106 where 15 of the *L*. *monocytogenes* isolates from this study was assigned, together with the isolates most closely related. (B) shows a subtree of SNP cluster PDS000025311.185 where two of the *L*. *monocytogenes* isolates from this study was assigned, together with the isolates most closely related according to this analysis

When picked up by the NCBI Pathogen Detection project the *L*. *monocytogenes* isolates in this study was assigned to two different SNP clusters, the group of 15 isolates was assigned to SNP Cluster PDS000032941.106 (393 isolates), while the group of two isolates was assigned to SNP Cluster PDS000025311.185 (1093 isolates). Figure 3 displays subtrees of the phylogenetic trees of these two SNP clusters with the isolates from this study together with the closest related isolates within the respective NCBI Pathogen Detection SNP cluster.

According to this analysis, the group of 15 isolates differs by a maximum of 4 SNPs from each other, while the two other isolates differ by only one SNP.

### 3.2 Analysis of 20 *Listeria* isolates with ON‐rep‐seq is in accordance with the WGS data regarding species level classification and strain level discrimination

#### 3.2.1 Species‐level classification

Classification of corrected reads from LCPs in 20 isolates identified 17 isolates as *L*. *monocytogenes* and three as *L*. *innocua*.

#### 3.2.2 Strain‐level discrimination

**FIGURE 4:**
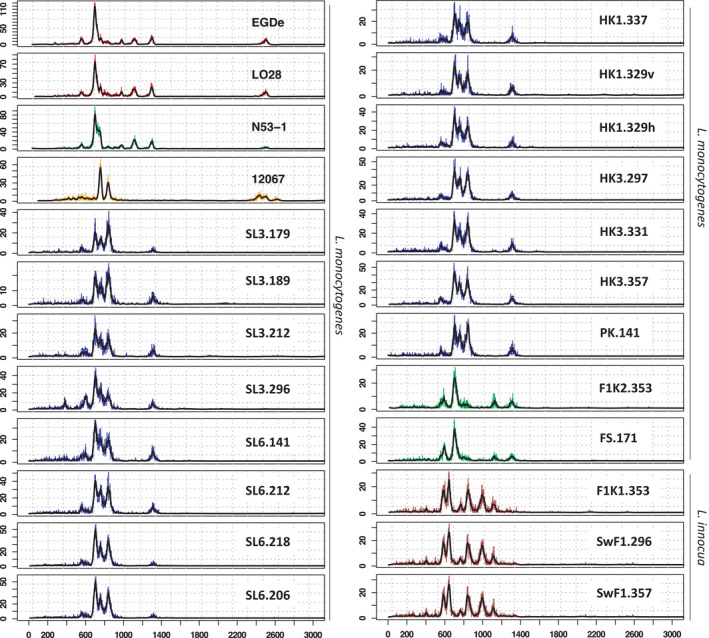
LCps (read length count profiles) generated from 20 putative *L*. *monocytogenes* isolates sampled from a salmon processing plant. The curves are a function of read length and abundance, where the position of the peak on the x‐axis corresponds to the length of the sequence and the height of the peaks corresponds to abundance. The four LCps at the top left are from reference strains analyzed in an earlier project (Krych et al., 2019). The two closely related strains EGD‐e and LO28 have previously been shown to be indistinguishable from each other than by SNP analysis. As is the case here as they have the same LCp. The two other strains N53‐1 and 12067 clearly show different profiles. Fifteen of the isolates analyzed in this study show the same LCp (blue) and are expected to be the same strain. Two of the isolates show an LCp (green) different from (c) but similar to each other, while three isolates show a third LCp (brown). This indicates that among the 20 isolates there are three different strains

**FIGURE 5:**
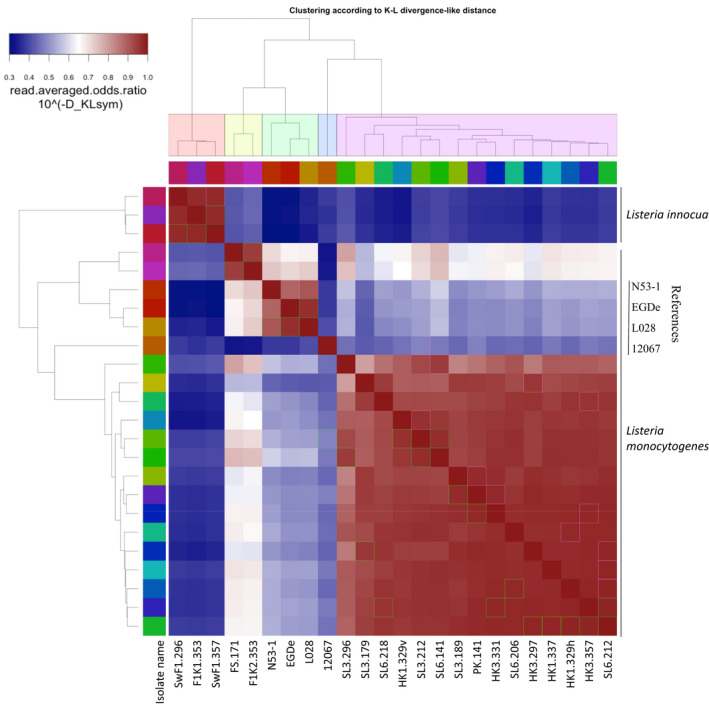
Heatmap showing the similarity (10^(‐D_KLsym)) between 20 isolates and 4 references, with clusters according to cut‐off=0.09. The three isolates (SwF1.296, SwF1.357, and F1K1.353) found to be *L*. *innocua* are clearly different from all the *L*. *monocytogenes* strains. The large group of 15 isolates with the same LCp cluster together, as do the two last isolates. This corresponds perfectly with the MLST classification based on WGS data

The read length count profiles (LCps) from the sequenced Rep‐PCR products identified three unique profiles among the selected isolates (Figures 4 and 5). Among 17 *L*. *monocytogenes* isolates two unique clusters of LCps were distinguished with two and 15 isolates (Figure 5). No differentiation in LCp profiles could be observed among three *L*. *innocua* isolates (Figure 4).

## 4 DISCUSSION

Species‐level and strain‐level discrimination of microorganisms is essential for a food processing plant to track microbial contamination sources in the value chain. Intra‐species variability exists in important characteristics such as virulence, pathogenicity, and drug resistance. During seven months a bacterial isolate can change due to environmental conditions, isolation, and culturing can generate new SNPs (Allard et al., 2012), and sequences from the same contamination source are most likely not identical even though they are of the same origin (Pightling et al., 2018).

In this study, a set of 20 putative *Listeria monocytogenes* isolates from a salmon processing plant were identified to species and differentiated down to strain level with ON‐rep‐seq and the results were evaluated by WGS. The isolates, originally detected through routine sampling in the processing plant, were selected from different time points and sampling points in the processing facility, with a focus on two gutting machines where *L*. *monocytogenes* had repeatedly been detected. The ON‐rep‐seq method separated the isolates into three distinct groups with unique LCps (read length count profiles). The taxonomic classification performed on the consensus reads from each peak revealed that these groups were two different *L*. *monocytogenes* strains and one *L*. *innocua* strain. This differentiation is in agreement with our former work where we described the relationship between unique LCps and associated strains (Krych et al., 2019). Testing novel methods on real industry case isolates is significant, and in this study, ON‐rep‐seq was able to unravel differences and similarities between the isolates. Results as unique LCps differentiating between strains, as presented here, will inform the quality control personnel at the processing plant that with high probability it is the same strain that caused the repeatedly positive tests in the gutting machines and head cutters. All the 15 *L*. *monocytogenes* isolates from the same area in the factory have the same LCp, while the two isolates from the filleting area have a different LCp and the *L*. *innocua* strains a third profile, and they all differ from the *L*. *monocytogenes* reference strains. The visualization of the strain differentiation in a heat map allows for an easy and intuitive interpretation of strain similarity. However, the classification in the ON‐rep‐seq method cannot identify exactly which strains they are unless they can be compared with identical LCps from a larger set of strains in a database.

So, WGS has currently the highest discrimination power as to resolution compared to other molecular typing techniques. However, making use of the power in this technology requires a high level of bioinformatic competence and computer infrastructure. Several commercial units provide a standardized or custom set of data analyses, yet this approach requires initial knowledge on tested organisms to customize the analysis. An increasing number of online web tools, free or paid, and several commercial softwares are also available, which all have their pros and cons (Jagadeesan, Baert, et al., 2019; Quainoo et al., 2017). In 2017 PulseNet International published their vision that WGS should be used by all public health laboratories to identify, characterize and subtype food pathogens for better and more accurate source tracking (Nadon et al., 2017). In the aftermath of this, the use of WGS among food companies was discussed in an industry workshop in 2019 (Amézquita et al., 2020). One of the barriers discussed was the development of expertise in sequencing and bioinformatics that is necessary, as well as the concern for the requirement of computer infrastructure and data storage needed (Amézquita et al., 2020).

Based on the WGS data, the isolates in this study were further characterized into sequence types (MLST). In correspondence with the identical LCps from the ON‐rep‐seq analysis, the group of two identical *L*. *monocytogenes* strains was identified as ST8 and the group of 15 *L*. *monocytogenes* strains as ST37.

The two isolates of *L*. *monocytogenes* ST8 were originally detected in the filleting area, the first isolate, FS.171, from salmon fillet and the second isolate, F1K2.353, in a filleting machine six months later. Strain ST8 has earlier been linked to a multi‐country outbreak of listeriosis in Denmark, Germany, and France in 2015–2018 which was due to the consumption of salmon products (EFSA, 2018). In addition, ST8 has been identified repeatedly over three years in a salmon processing plant in Denmark (Schmitz‐Esser et al., 2015). In Norway, *L*. *monocytogenes* ST8 has been frequently detected in one salmon slaughterhouse for 13 years (Fagerlund et al., 2016). All this demonstrates that *L*. *monocytogenes* of this ST can be persistent, and it can cause listeriosis. *L*. *monocytogenes* ST37 has been detected in both food products and food processing environments associated with meat, dairy, and vegetables, respectively (Cabal et al., 2019; Stessl et al., 2020; Tomáštíková et al., 2019). It is however suspected to be a less persistent strain than ST8 (Muhterem‐Uyar et al., 2018).

The phylogenetic analyses done, both by CSI Phylogeny and NCBI Pathogen Detection confirms the grouping of the isolates demonstrated by ON‐rep‐seq. CSI Phylogeny SNP tree in Figure 1 indicates that the *L*. *monocytogenes* isolates cluster in two different groups in exact accordance with the ON‐rep‐seq LCps, and the MLST sequence type, additionally, both groups are somewhat different from the reference strain *L*. *monocytogenes* EGD‐e. The three *L*. *innocua* strains are not included in the SNP phylogenetic tree as their relationship to the *L*. *monocytogenes* are too distinct.

In NCBI Pathogen Detection phylogenetic tree the two groups of isolates are assigned to two different SNP clusters which means that the groups differ by >50 SNPs. Within the group of 15 isolates, the isolates differed by a maximum of 4 SNPs while there was only one SNP difference between the two isolates in the small group. This supports the conclusion that all the isolates within each group are the same strain and that we are dealing with two strains of *L*. *monocytogenes* in this material. The minimum SNP difference to a clinical isolate is 25–29 SNPs and the closest environmental isolate is an isolate from a dairy barn in Finland from 2015 with a 27–31 SNP difference. Within this SNP cluster, there are no other isolates with a registered association to salmon, only four isolates associated with fish (herring) and one isolate associated with seafood, namely a seafood factory in Ireland. There is a group of five isolates associated with food processing environment collected in the UK in 2011 with a minimum SNP difference of 34.

The two isolates (FS.171 and F1K2.353) assigned to SNP Cluster PDS000025311.185 belong to ST8. In this analysis the minimum SNP difference from our isolates to a clinical isolate, namely from a case of human listeriosis in Germany in 2018, is 25 and 26 SNPs. Within this SNP cluster there are 15 other isolates associated with salmon, mostly smoked salmon. Three of these come from salmon processing facilities in Norway, the closest being 32–33 SNPs different from the isolates in this study. Five additional isolates in this SNP cluster were associated with fish or seafood and 11 isolates were reported to come from the processing environment.

The average nucleotide identity (OrthoANI values) indicated a high degree of conservation among the different isolates. In this analysis, all the 15 isolates of ST37 had an ANI value to each other of 100.00% which indicates that they are most likely the same strain. Considering an ANI cutoff value of <99% to differentiate between strains, these strains cannot be differentiated with the ANI index. The two ST8 isolates share an ANI value of 99.97% and are by this method considered to be the same strain. The ANI values between strains of different ST were all >99.00%, which means that none of the *L. monocytogenes* isolates in this study can be differentiated from each other by this method.

None of the *L*. *monocytogenes* isolates in this study carried a truncated *inlA* gene. The virulence factor internalin A in *L*. *monocytogenes*, encoded by *inlA*, plays a critical role in crossing the intestinal barrier to give a systemic infection in humans (Olier et al., 2005). Clinical isolates of *L*. *monocytogenes* usually carry a fully functional *inl*A gene (Gorski et al., 2016). Different mutations in this gene can lead to premature stop codons (PMSC) (Van Stelten et al., 2010) and have been identified in 45%–50% of food isolates analyzed (Upham et al., 2019; Van Stelten et al., 2010). This can indicate a lower potential of pathogenesis (Olier et al., 2005) and this gene has been suggested as a genetic marker for risk assessment (Upham et al., 2019). In this study, all the *L*. *monocytogenes* isolates carried a full length and predictably fully functional *inl*A gene meaning that they must be considered as a severe risk for human infection if they contaminate the food product.

In the analysis of pathogenicity done with PathogenFinder, all the isolates, including *L*. *innocua*, were predicted to be human pathogens. However, the *prf*A gene, coding for positive regulatory factor (PrfA) of *L*. *monocytogenes*, was not present in the *L*. *innocua* isolates. This factor regulates and activates most of the known virulence genes by binding to a palindromic *prf*A recognition sequence located in the promoter region (Glaser et al., 2001; Greene & Freitag, 2003). This means that many of the genes involved in pathogenesis will not be expressed in these isolates even though they are present and therefore these isolates are probably not pathogenic. The *prf*A gene was present in all *L*. *monocytogenes* isolates in full length and with 100% identity to the reference gene.

In this study, the isolates used for analysis were selected based on when and where they were detected in the processing plant, and in connection to the area with frequent *Listeria* detection. The analyses done revealed a low diversity in the tested isolates and thereby give a limited base for a thorough evaluation of the ON‐rep‐seq method. However, it shows that in this specific industry case it was indeed the same strain (group of 15 similar isolates) causing the repeatedly positive tests in the two gutting machines and downstream equipment. All the tested isolates were detected throughout seven months. This is a relatively short time to evaluate if the strains are persistent strains or transient strains. However, the result from this study supports that there is a strain that persists in equipment and environment in the processing plant for these seven months.

Daily, many industries cannot afford long‐term studies on strain persistence, and the main information regarding putative contaminations is limited to the species level typing, namely the presence/absence of *L*. *monocytogenes*. In cases where strain tracing is necessary e.g. in presence of frequent positive tests for *Listeria* in certain areas or equipment, swift preventative action is needed followed by the validation of the action. In such cases, a fast, reliable, and cost‐effective approach is desirable.

For routine analysis of *Listeria*, many companies use a two‐step method, first iQ‐Check^TM^ *Listeria* spp. PCR Detection Kit, secondly, positive samples plated on Rapid’L.mono agar plates. On these plates, *L*. *monocytogenes* usually appear as blue/green colonies with no colored halo whereas e.g *L*. *innocua* appear as white colonies. There are exceptions, however, where white colonies were identified as *L*. *monocytogenes*, and blue colonies were confirmed as *L*. *innocua*. (Greenwood et al., 2005), as was also the case for some of the isolates in this study. Those three isolates (F1K1.353, SwF1.296, and SwF1.357) did not show lecithinase activity when grown on BLA, which indicates that they are not *L*. *monocytogenes* but another *Listeria* species. However, the isolates were not excluded from the study based on this considering they had been identified as *L*. *monocytogenes* by the company's analysis on Rapid’L.mono agar and when grown on Rapid’L.mono in our laboratory the inconclusive morphology (areas with blue halo) was confirmed. It was therefore of interest to get a thorough analysis of these isolates as well. The identification of *L*. *innocua* in this study highlights the difficulty for the processing plant to correctly differentiate *Listeria* even at the species level with the methods available.

Many companies have established a comprehensive test regime to detect and eliminate *L*. *monocytogenes* from their value chain and this system can include storage of presumptive *L*. *monocytogenes* isolates in case of tracking and tracing of source contamination. It must be acknowledged that; the more they test – the more they find, and for some processing plants, this can lead to several hundred isolates a year. Performing WGS on hundreds of isolates is not applicable due to the costs, workload, data processing, and data storage needed (Amézquita et al., 2020; Jagadeesan, Gerner‐Smidt, et al., 2019). Sequencing a small number of isolates in a tracing situation will be the most likely scenario but selecting the most representative isolates for this might be a challenge. As demonstrated here the ON‐rep‐seq method gives sufficient information for preliminary source tracking of pathogens in the food industry to serve as a screening method before doing WGS and can in some cases even serve as an alternative method to WGS.

ON‐rep‐seq as a fast‐screening method offers much more accurate taxonomic identification than 16S rRNA gene sequencing with simultaneous access to a strain level discrimination comparable to that obtained from the WGS. Table 5 lists some commercial prices for different traditional typing methods and compares them to sequencing‐based methods as Sanger sequencing of 16S rRNA gene, WGS, and the novel method ON‐rep‐seq. This overview shows that the cost of ON‐rep‐seq is within the same range as that of Sanger sequencing, making it 8 to 10 times more cost‐effective than the alternative typing methods delivering similar information regarding identification and differentiation. The method can be introduced to facilities at a very low cost since the MinION sequencing platform is available at about $1000. Furthermore, the possibility for analysis of up to 96 isolates on a Flongle, which is the cheapest flow cell available so far ($90), ensures low running costs with the highest resolution level that offers comparable resolution to WGS in terms of classification.

**TABLE 5:** Comparison of commercial prices for traditional typing methods, 16S sequencing, WGS, and ON‐rep‐seq

Typing method	Unit price (1–10 units/next 10)	Additional preparation costs (DNA‐extraction, QC)	Additional one‐time cost	Total cost for 20 isolates
PFGE	170 €/89 €			2590 €^a^
Serotyping	180 €/117 €			2970 €^a^
WGS	85 €	2 €	27 €	1767 €^a^
16S (Sanger seq)	4 €	4 €		160 €^a^
ON‐rep‐seq	10 €~2 €^c^	4 €		280 €^b^

^a^ Commercial prices.

^b^ Estimated price based on the price for one Flongle, library preparation, and necessary working hours.

^c^ The price for each sample if 96 samples are analyzed simultaneously on the Flongle.

## 5 CONCLUSION

With this study, we demonstrate that the recently developed fingerprinting method combined with nanopore sequencing called ON‐rep‐seq is a promising, rapid, cost‐effective, and less laborious alternative to the whole genome sequencing for species‐level identification and strain level discrimination of *Listeria* species.

From a set of 20 isolates, 17 *L*. *monocytogenes* and 3 *L*. *innocua* were identified and the *L*. *monocytogenes* isolates were further differentiated into two strains. The analysis done on WGS data showed the same, and no further differentiation of the isolates was obtained.

The material in this study is however very limited. To evaluate the discriminatory power of ON‐rep‐seq more thoroughly a more diverse set of isolates will be necessary.

## CONFLICT OF INTEREST

None declared.

## AUTHOR CONTRIBUTIONS

**Gunn Merethe Bjørge Thomassen:** Conceptualization (equal); Formal analysis (equal); Writing‐original draft (lead); Writing‐review & editing (equal). **Lukasz Krych:** Conceptualization (equal); Formal analysis (equal); Writing‐review & editing (equal). **Susanne Knøchel:** Writing‐review & editing (equal). **Lisbeth Mehli:** Conceptualization (equal); Writing‐original draft (supporting); Writing‐review & editing (equal).

## ETHICS STATEMENT

None required.

## Data Availability

Sequence reads from whole‐genome sequencing and ON‐rep‐seq are available at the NCBI repository under the BioProject number PRJNA763206: https://www.ncbi.nlm.nih.gov/bioproject/PRJNA763206. The ON‐rep‐seq data analysis toolbox is available on GitHub: https://github.com/lauramilena3/On‐rep‐seq and Zenodo: https://doi.org/10.5281/zenodo.3384841
